# First person – Sabrina Alam

**DOI:** 10.1242/dmm.049668

**Published:** 2022-06-23

## Abstract

First Person is a series of interviews with the first authors of a selection of papers published in Disease Models & Mechanisms, helping early-career researchers promote themselves alongside their papers. Sabrina Alam is first author on ‘ *Snrpb* is required in murine neural crest cells for proper splicing of genes essential for craniofacial morphogenesis’, published in DMM. Sabrina is a PhD student in the lab of Dr Loydie A. Jerome-Majewska at McGill University, Montreal, Canada, investigating the role of splicing in the complexity of craniofacial development.


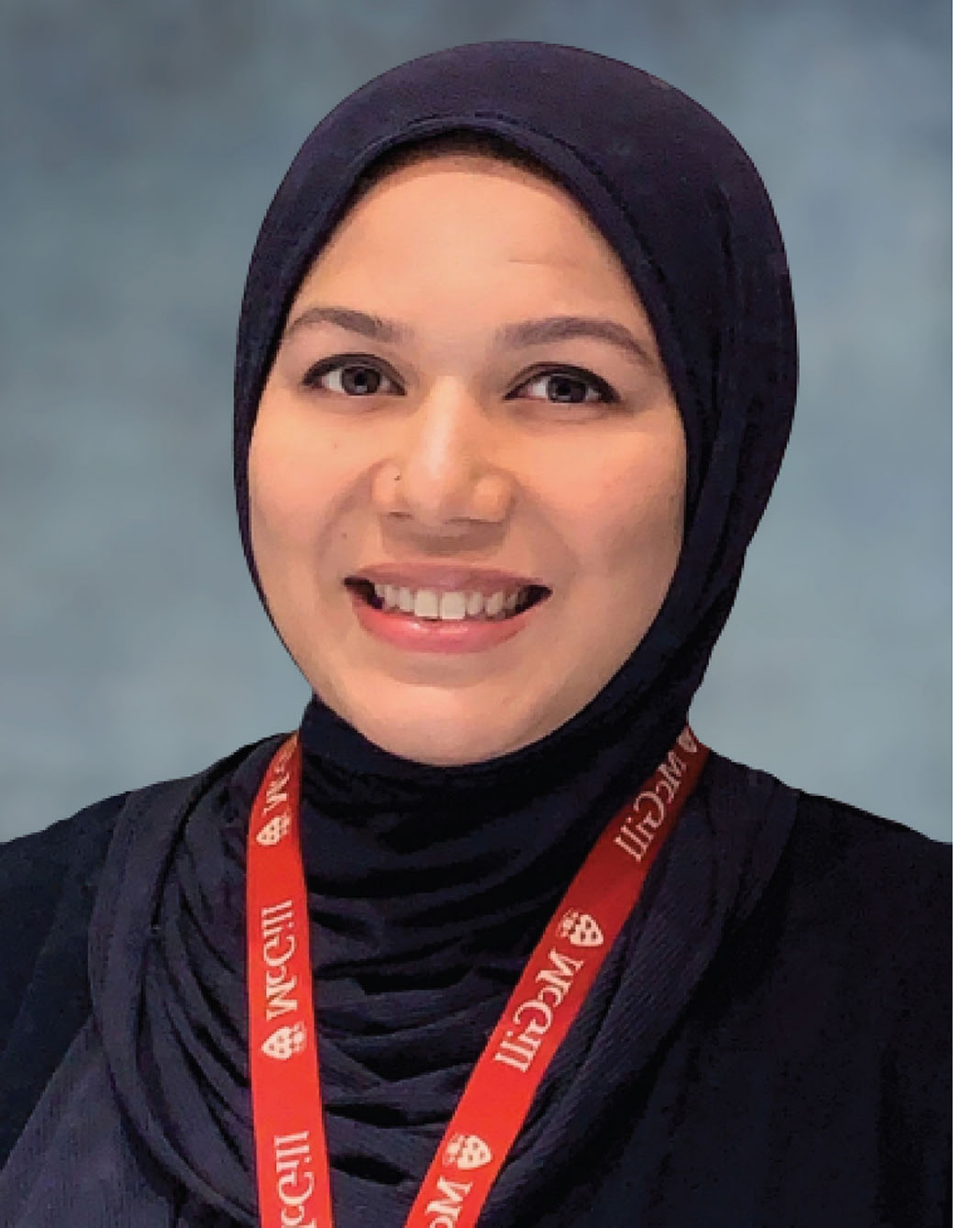


**Sabrina Alam**

**How would you explain the main findings of your paper to non-scientific family and friends?**

Cerebrocostomandibular syndrome (CCMS) is a rare disorder that affects one in a million births. CCMS patients mostly have abnormal development of the head, face and ribs. It is known that a gene named *SNRPB* is mutated in CCMS. *SNRPB* is required in every cell for a vital molecular event called transcription in which messenger RNA is produced, which carries the information from DNA, which is then translated into protein. *SNRPB* is needed everywhere, but why mutations in *SNRPB* cause specific abnormalities of the head and face in CCMS patients is unknown. So, we aimed to understand the molecular mechanisms of the craniofacial abnormalities seen in CCMS by using the mouse as a disease model, because of the genetic and developmental similarities between humans and mice. We created a mutation in the *Snrpb* gene in mice and recapitulated craniofacial abnormalities that are found in CCMS patients. We found that, when *Snrpb* was deleted, several genes that play key roles in craniofacial development were affected. Thus, to understand the role of *SNRPB* during craniofacial development and how its mutation results in the abnormalities seen in CCMS patients, we generated the very first animal model to provide new insights into CCMS.

“[…] we have provided a valuable tool for better understanding of CCMS and shed light on how disrupted splicing due to *Snrpb* mutation can result in molecular cascades that affect craniofacial development.”

**What are the potential implications of these results for your field of research?**

In our work, we have developed the first animal model for CCMS and have shown the development-specific role of *Snrpb* during morphogenesis. We performed transcriptome analyses of the *Snrpb* mutants and showed several transcripts that are essential for craniofacial development that are affected, which could potentially be responsible for the abnormal development of craniofacial structures in CCMS. As knowledge of the role of splicing factors in development is still emerging and the molecular mechanisms of CCMS are not understood, we have provided a valuable tool for better understanding of CCMS and shed light on how disrupted splicing due to *Snrpb* mutation can result in molecular cascades that affect craniofacial development.

**What are the main advantages and drawbacks of the model system you have used as it relates to the disease you are investigating?**

The craniofacial structures and their development are remarkably similar between mice and humans. The complexity and coordination of diverse types of cells and tissues to form the craniofacial structures happening in days in mice are parallel to those happening in months in humans. Thus, the model gives the advantage of studying craniofacial disorders in mice, especially for a rare disease like CCMS. However, mouse models are often criticised for not always accurately mimicking the human disease phenotypes. For example, we found our mice do not survive in *Snrpb* heterozygous condition while CCMS patients are all heterozygous. One limitation of our model for CCMS is, we could not make the same mutation in *Snrpb* that is found in the patients. The mutation occurs in an alternative exon 2 of the gene that is thought to reduce the overall level of SNRPB. We made our model by reducing the SNRPB level, but it did not have the exact point mutation in the same alternative exon 2 in mice.

**Figure:**
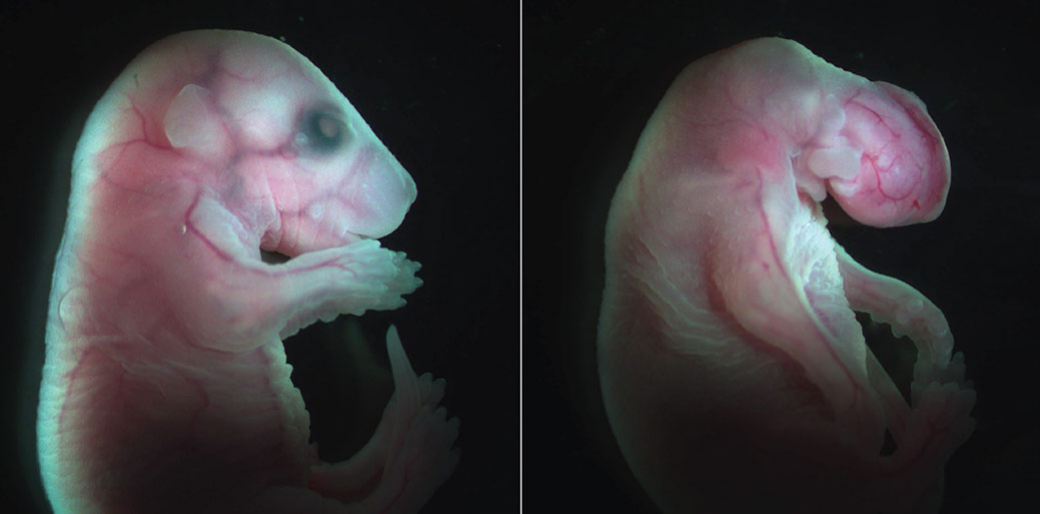
Snrpb is required for craniofacial development. On the right, a 17.5-day-old embryo mutant for *Snrpb* in the neural crest cells, showing severe craniofacial abnormalities with no face formed. The embryo on the left is a normal one.

**What has surprised you the most while conducting your research?**

While conducting my research, I was fascinated each time I looked under the dissection microscope and appreciated how precisely and beautifully the embryos develop.

**Describe what you think is the most significant challenge impacting your research at this time and how will this be addressed over the next 10 years?**

CCMS is a rare congenital disorder and, like for other rare disease research, studying CCMS is not simple because there are not a lot of patients reported for this disease. It is challenging to collect and correlate patient data with what we find in the lab. Building collaborations with other scientists and doctors will be the key to addressing this issue. The other challenge my research is facing is the lack of available funding. I assume major funding agencies give preference to research that is likely to have a direct impact on patients. I think raising awareness is important for rare disease research and to convince funding bodies that the research can lead to the discovery of other components that may be affecting common diseases and support the development of effective medications.

**What changes do you think could improve the professional lives of early-career scientists?**

I think the professional life of early-career scientists is not straightforward. Funding is one of the huge barriers for many who look to make their next steps. Finding the right job is also a challenge for many. Often PhD students find it difficult to make a choice after their graduation. In this case, mentors could particularly play a role by individually tailoring advice on career options. Job instability is also an issue in many places. Many talented early-career scientists feel hopeless because of the growing pressure of getting funding when they are non-tenured. Research is not a 9-to-5 job and needs a lot of time, effort and devotion. I think it is particularly important to recognise and support the development of researchers. Funding should be adequately distributed with importance to every field. The working environments of scientists should be ambient so that it is possible to keep it balanced with life. I also think more inclusive programs should be navigated around the globe for early-career scientists as they usually come up with the latest ideas and approaches. However, I appreciate the growing networking opportunities that are becoming available because of social media.

“[…] efficient science requires passion.”

**What's next for you?**

In the coming few months, I will submit and defend my PhD thesis. I am interested in doing a postdoctoral fellowship after graduation. I would love to pursue a career in academia. I am passionate about science, and I believe efficient science requires passion. I know it will be a bit challenging because academia is highly selective and there is an oversupply of talented graduates. I am glad that my supervisor always inspires and advises me on what I want to do for my career.
